# Involvement of Endocytosis in the Transdermal Penetration Mechanism of Ketoprofen Nanoparticles

**DOI:** 10.3390/ijms19072138

**Published:** 2018-07-23

**Authors:** Noriaki Nagai, Fumihiko Ogata, Miyu Ishii, Yuya Fukuoka, Hiroko Otake, Yosuke Nakazawa, Naohito Kawasaki

**Affiliations:** 1 Faculty of Pharmacy, Kindai University, 3-4-1 Kowakae, Higashi-Osaka, Osaka 577-8502, Japan; ogata@phar.kindai.ac.jp (F.O.); 1833420012r@kindai.ac.jp (M.I.); 1833420015f@kindai.ac.jp (Y.F.); hotake@phar.kindai.ac.jp (H.O.); kawasaki@phar.kindai.ac.jp (N.K.); 2 Faculty of Pharmacy, Keio University, 1-5-30 Shibakoen, Minato-ku, Tokyo 105-8512, Japan; nakazawa-ys@pha.keio.ac.jp

**Keywords:** nanoparticle, endocytosis, transdermal delivery system, ketoprofen, pharmacological inhibitor

## Abstract

We previously designed a novel transdermal formulation containing ketoprofen solid nanoparticles (KET-NPs formulation), and showed that the skin penetration from the KET-NPs formulation was higher than that of a transdermal formulation containing ketoprofen microparticles (KET-MPs formulation). However, the precise mechanism for the skin penetration from the KET-NPs formulation was not clear. In this study we investigated whether energy-dependent endocytosis relates to the transdermal delivery from a 1.5% KET-NPs formulation. Transdermal formulations were prepared by a bead mill method using additives including methylcellulose and carbopol 934. The mean particle size of the ketoprofen nanoparticles was 98.3 nm. Four inhibitors of endocytosis dissolved in 0.5% DMSO (54 μM nystatin, a caveolae-mediated endocytosis inhibitor; 40 μM dynasore, a clathrin-mediated endocytosis inhibitor; 2 μM rottlerin, a macropinocytosis inhibitor; 10 μM cytochalasin D, a phagocytosis inhibitor) were used in this study. In the transdermal penetration study using a Franz diffusion cell, skin penetration through rat skin treated with cytochalasin D was similar to the control (DMSO) group. In contrast to the results for cytochalasin D, skin penetration from the KET-NPs formulation was significantly decreased by treatment with nystatin, dynasore or rottlerin with penetrated ketoprofen concentration-time curves (*AUC*) values 65%, 69% and 73% of control, respectively. Furthermore, multi-treatment with all three inhibitors (nystatin, dynasore and rottlerin) strongly suppressed the skin penetration from the KET-NPs formulation with an *AUC* value 13.4% that of the control. In conclusion, we found that caveolae-mediated endocytosis, clathrin-mediated endocytosis and macropinocytosis are all related to the skin penetration from the KET-NPs formulation. These findings provide significant information for the design of nanomedicines in transdermal formulations.

## 1. Introduction

Ketoprofen is a non-steroidal anti-inflammatory drug (NSAID) that is scarcely soluble in water with a log partition coefficient are 0.185 mg/mL, 3.11, respectively. Ketoprofen mechanism of action involves the reversible inhibition of cyclooxygenase (COX), which, in turn, blocks the synthesis of prostaglandins (PGs) from arachidonic acid. Ketoprofen has been utilized extensively for the treatment of rheumatoid arthritis and associated diseases, since elevated levels of PGs sensitize peripheral nociceptors and exacerbate painful stimuli [1,2]. On the other hand, the oral administration of ketoprofen, as with other NSAIDs, has undesirable side effects on the gastrointestinal tract [3,4]. One promising method to reduce the adverse effects is to deliver the drug through the skin. However, due to the excellent barrier function of the skin, the need to use safe and effective enhancers to improving the transdermal absorption of drugs is well recognized [5,6,7,8]. In an attempt to improve the skin permeation characteristics of ketoprofen, various methods to enhance the delivery of ketoprofen through the skin, such as liposomes, incorporation of penetration enhancers, microneedles, transdermal patches, and microemulsions have been investigated [9,10,11,12]. Of particular promise are transdermal delivery systems using nanoparticles [9,10,11,12]. The properties of nanoparticles play a significant role in determining their final biological fate [13,14,15], with size, shape, surface functionality and stiffness being the most important design parameters for nanoparticle-mediated drug carriers [13,16]. For instance, nanoparticles with diameters of 60–100 nm have been demonstrated to be of the optimal size for the cellular uptake process [17,18,19]. Therefore, we designed transdermal formulations containing ketoprofen nanoparticles (KET-NPs formulation, particle size approximately 80–200 nm), and showed a high accumulation of ketoprofen released from the KET-NPs formulation in skin tissues. Further, the therapeutic effect on inflammation of the KET-NPs formulation is significantly greater than that of commercially available ketoprofen ointments (dissolution type) [20]. The design of a KET-NPs formulation may represent a novel transdermal delivery system for the management of inflammation. However, the transport pathways for drugs from transdermal formulations containing nanoparticles remain unclear. Therefore, it is important to understand the mechanism of transdermal penetration from the KET-NPs formulation.

Nanoparticles do not enter cells simply via diffusion, and the relationship between endocytosis and nanoparticle-based drug delivery has been revealed by many researchers [21,22,23,24,25]. The pathways of uptake of nanoparticles can be divided into phagocytosis/pinocytosis, and passive penetration. Phagocytosis is performed by specialized cells such as macrophages, and plays a role in the clearance of particles with diameters greater than 0.5 μm. On the other hand, smaller particles can be taken up by pinocytosis, which can be further classified into clathrin-mediated endocytosis (CME), caveolae-mediated endocytosis (CavME), and macropinocytosis (MP) [26,27]. CME is used by all mammalian cells, representing an important part of cellular entry [27]. The size of vesicles taken up by CME is about 100 nm. CavME is likewise a common pathway for cellular entry exploited by particles 60–80 nm in size [28]. This pathway bypasses lysosomes [28], and is the pathway exploited by many pathogens including viruses and bacteria to avoid lysosomal degradation [29]. MP is an actin-regulated process that involves the engulfment of a large quantity of extracellular fluid and particles by plasma membrane ruffling. These membrane ruffles exhibit different shapes, and when closed, form large organelles called macropinosomes [30]. MP covers a broad range of particle sizes from 100 nm to 5 μm [31,32,33].

In this study, we investigated whether these forms of endocytosis are related to the skin penetration of ketoprofen from KET-NPs formulations using pharmacological inhibitors.

## 2. Results

### 2.1. Evaluation of the Release of Ketoprofen Nanoparticles from KET-NPs Formulation

**Figure 1:**
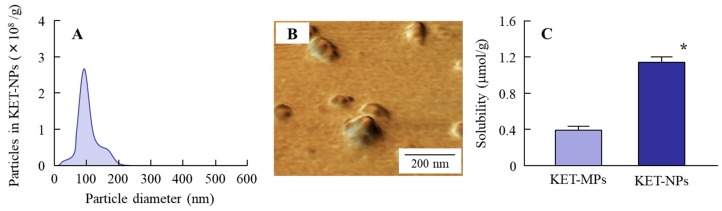
Particle size frequencies (**A**); SPM images (**B**) and solubility (**C**) of ketoprofen particles in the KET-NPs formulation. Mean ± S.E. *n* = 6. * *p* < 0.05 vs. KET-MPs formulation. The particle size of ketoprofen in the KET-NPs formulation was 98.3 ± 48.7 nm, and the ratio of nanoparticles to solubilized ketoprofen was 98%.

**Figure 2:**
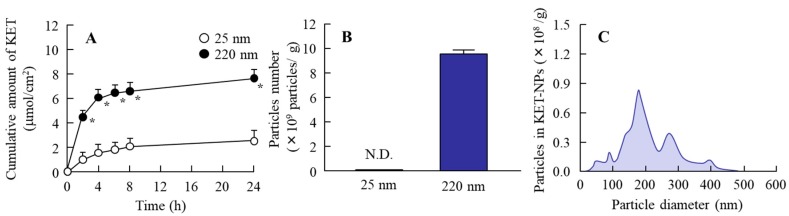
Ketoprofen release from the KET-NPs formulation through 25 nm and 450 nm pore membranes. (**A**) Drug release from the KET-NPs formulation through the membranes; (**B**) Number of ketoprofen particles released from the KET-NPs formulation; (**C**) Particle size frequencies of ketoprofen released from the KET-NPs formulation 24 h after application in the 450 nm pore membrane. The ketoprofen in the Franz diffusion cell (reservoir chamber filled with purified water) after the application of the KET-NPs formulation was measured by HPLC, and the number of particles was counted using NANOSIGHT LM10. Means ± S.E. *n* = 6. N.D., not detectable. * *p* < 0.05 vs. 25 nm-pore membrane for each category. Ketoprofen was released from the KET-NPs formulation in the nanoparticle state (mean particle size, 189.3 ± 24.5 nm).

Figure 1 shows the size frequency, a scanning probe microscopic (SPM) image and solubility of the ketoprofen particles in the KET-NPs formulation. Bead mill treatment decreased the size of ketoprofen. The particle size in the 1.5% KET-NPs formulation was 98.3 ± 48.7 nm, with 1.51 × 10^10^ ± 0.04 × 10^10^ ketoprofen particles/g. Although the amount of dissolved ketoprofen (1.15 ± 0.05 μmol/g, *n* = 6) was also enhanced in comparison with the KET-MPs formulation (0.39 ± 0.05 μmol/g, *n* = 6), the amount dissolved ketoprofen in the KET-NPs formulation remained low with 98% of the ketoprofen in the nanoparticle state. Figure 2 shows profiles for the release of ketoprofen particles from the KET-NPs formulation. Ketoprofen release from the KET-NPs formulation through a 450 nm pore size membrane was significantly higher than through a 25 nm pore size membrane. The number of ketoprofen nanoparticles was also enhanced in the reservoir chamber. In the 24 h after application, 9.6 ± 0.3 × 10^9^ particles/g were detected in the reservoir chamber, and the particle size frequency of released ketoprofen nanoparticles remained in the nano order (particle size 189.3 ± 24.5 nm).

### 2.2. Effect of Energy Dependent Endocytosis on the Transdermal Delivery of Ketoprofen Nanoparticles in the KET-NPs Formulation

**Figure 3:**
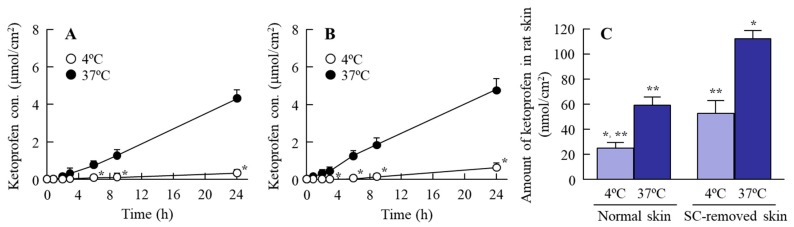
Transdermal penetration of ketoprofen released from the KET-NPs formulation at 4 °C and 37 °C. (**A**) Penetration of the KET-NPs formulation through skin containing SC (normal skin); (**B**) Penetration of the KET-NPs formulation through skin from which the SC was removed (SC-removed skin); (**C**) Amount of ketoprofen in rat skin 24 h after treatment the KET-NPs formulation at 4 °C and 37 °C. The SC was removed by tape stripping. The KET-NPs formulation was applied to skin with (normal skin) or without SC (SC-removed skin). Mean ± S.E. *n* = 6. * *p* < 0.05 vs. normal skin at 37 °C for each category. ** *p* < 0.05 vs. SC-removed skin at 37 °C for each category. The transdermal penetration and amount of ketoprofen in the SC-removed skin was higher than in normal skin. In addition, the transdermal penetration and accumulation of the drug into skin was prevented under the 4 °C conditions in both normal and SC-removed skin.

Figure 3 shows transdermal penetration profiles for ketoprofen particles from the KET-NPs formulation under conditions of inhibited energy-dependent endocytosis (4 °C) and under normal conditions (37 °C); Table 1 summarizes the pharmacokinetic parameters estimated from the data for the in vitro transdermal penetration shown in Figure 3A,B. The penetration profile for ketoprofen through the stratum corneum (SC)-removed skin was greater than through normal skin, and the penetration rate (*J*_c_) for normal skin was higher than for SC-removed skin at 37 °C. In addition, the accumulation of ketoprofen in SC-removed skin was higher than in normal skin at both 4 °C and 37 °C (Figure 3C). Moreover, no ketoprofen nanoparticles were detected in the reservoir chamber after application of the KET-NPs formulation to skin with or without SC (the number of ketoprofen nanoparticles was below the detection limit of the NANOSIGHT LM10). On the other hand, the penetration of ketoprofen was significantly prevented at 4 °C, with the amount penetrated (*AUC*_Penetration_) only 12.2% that at 37 °C 24 h after the application of KET-NPs formulation to normal skin.

### 2.3. Determination of the Endocytosis Pathway for Ketoprofen Nanoparticles Using Pharmacological Inhibitors

**Figure 4:**
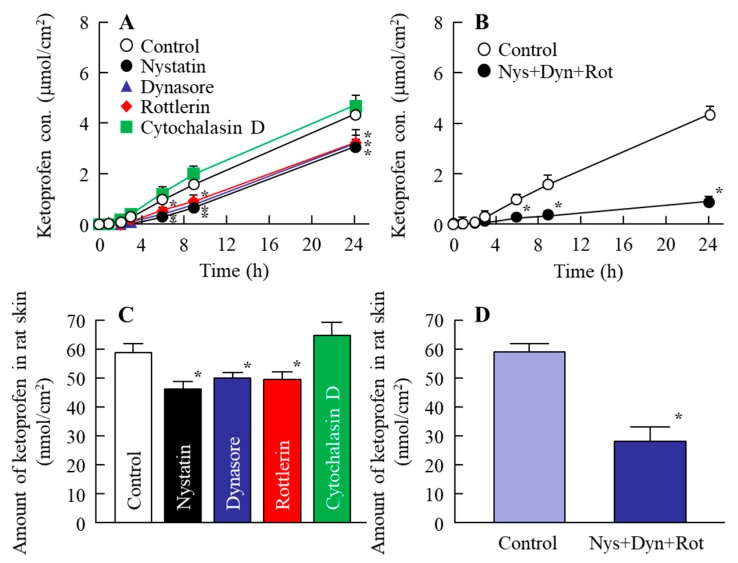
Effect of endocytosis on the transdermal penetration of ketoprofen released from the KET-NPs formulation. (**A**) Effect of endocytosis inhibitors (nystatin, dynasore, rottlerin and cytochalasin D) on the penetration from the KET-NPs formulation through the skin; (**B**) Changes in the transdermal penetration in the KET-NPs formulation by multi-treatment with three inhibitors (nystatin, dynasore and rottlerin; Nys + Dyn + Rot); (**C**) Effect of endocytosis inhibitors (nystatin, dynasore, rottlerin and cytochalasin D) on the amount of ketoprofen in rat skin 24 h after application of the KET-NPs formulation; (**D**) Changes in drug accumulation from the KET-NPs formulation by multi-treatment with three inhibitors (nystatin, dynasore, rottlerin; Nys + Dyn + Rot). The ketoprofen concentration in rat skin was measured 24 h after application of the KE-NPs formulation. Mean ± S.E. *n* = 6–8. * *p* < 0.05 vs. control for each category. The transdermal penetration of ketoprofen nanoparticles was promoted by the additive effects of CavME, CME and MP.

**Figure 5:**
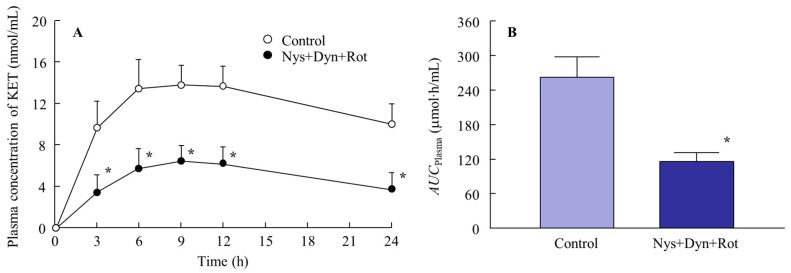
Effect of endocytosis on the percutaneous absorption of ketoprofen released from the KET-NPs formulation. (**A**) Changes in percutaneous absorption from the KET-NPs formulation by multi-treatment with three inhibitors (nystatin, dynasore and rottlerin; Nys + Dyn + Rot); (**B**) Effect of multi-treatment with three inhibitors (nystatin, dynasore and rottlerin; Nys + Dyn + Rot) on *AUC*_Plasma_ in rats treated with the KET-NPs formulation. Mean ± S.E. *n* = 6. * *p* < 0.05 vs. control for each category. The percutaneous absorption of from the KET-NPs formulation was attenuated by the inhibitory effects of CavME, CME and MP.

Figure 4 shows the changes in the penetration and accumulation of ketoprofen particles from the KET-NPs formulation into skin treated with endocytosis inhibitors; Table 2 summarizes the pharmacokinetic parameters estimated from the data for the in vitro transdermal penetration shown in Figure 4A,B. Although, the transdermal penetration into skin treated with cytochalasin D was similar to that of the control, treatment with nystatin, rottlerin or dynasore all significantly decreased the transdermal penetration of the KET-NPs formulation. Moreover, the amount of ketoprofen in the skin tissue following the application of the KET-NPs formulation also lower than in the control group by treatment of nystatin, rottlerin or dynasore. In addition, the *AUC*_Penetration_ and accumulation in skin treated with all three endocytosis inhibitors (nystatin, rottlerin and dynasore) were similar to those at 4 °C (inhibited energy-dependent endocytosis) as shown in Table 1 and Figure 3. Figure 5 shows the changes in the percutaneous absorption of the KET-NPs formulation in rats multi-treated with nystatin, rottlerin and dynasore. The percutaneous absorption of the KET-NPs formulation was significantly decreased by treatment with the three inhibitors, and the *AUC*_Plasma_ in rats multi-treated with the three inhibitors was 13.4% that of the control.

## 3. Discussion

Ketoprofen is a widely prescribed NSAID for the management of pain from inflammation, such as in arthritis, but oral administration produces systemic adverse effects including gastrointestinal irritation. Since the gastrointestinal side effects can be avoided by using transdermal formulations, the development of a drug delivery system that improves the transdermal absorption of the drug is expected. We previously reported that transdermal formulations containing ketoprofen solid nanoparticles (KET-NPs formulation) provide high skin permeability and drug accumulation, and that the anti-inflammatory effects of the KET-NPs formulation are significantly greater than those of commercially available ketoprofen ointments (dissolution type) in adjuvant-induced arthritic rats [20]. However, the transport pathway remains unclear for the KET-NPs formulation. In this study, we investigated the effect of endocytosis on the skin penetration of the KET-NPs formulation, and found that three endocytosis pathways (CavME, CME and MP) are related to the skin penetration of ketoprofen nanoparticles.

The biological fate of the nanoparticles was determined by their properties [13,14,15]; specifically, size and shape are the most important factors in the transport pathways [13,16]. We demonstrated the size and shape of the nanoparticles in the KET-NPs formulation by NANOSIGHT LM10 and SPM imaging, respectively. It has previously been reported that nanoparticles with diameters in the range of 60–100 nm are optimal for the cellular uptake process [17,18,19]. Therefore, the KET-NPs formulation in this study may be suitable for the cellular uptake process, since the particle size in this formulation is 98.3 ± 48.7 nm (Figure 1A). Moreover, 4–7 of 15 nm-ketoprofen particles were aggregated, and each ketoprofen particle looks like a ledge on the surface of the KET-NPs formulation (Figure 1B). In addition, we determined the particle size of the ketoprofen particles released from the KET-NPs formulation in an in vitro drug release test using the Franz diffusion cell (Figure 2). The particle size of the released ketoprofen (9.58 ± 0.34 × 10^9^ particles/g) remained at the nano order (189.3 ± 24.5 nm, Figure 2C). These results show that ketoprofen from the KET-NPs formulation is released as nanoparticles rather than in a dissolved form. On the other hand, the ketoprofen particle size after penetration of the 450 nm pore membrane (Figure 2C) was larger than that in KET-NPs formulation (Figure 1A). These difference of particle size frequencies in the Figure 1A and Figure 2C may be caused by the aggregation, since the dispersible of ketoprofen nanoparticles was unstable in the receptor medium (purified water) used Franz diffusion cell.

Next, we investigated the transport pathways of ketoprofen nanoparticles into rat skin. It is known that the SC has a barrier function in the skin to prevent particles from penetrating into the skin. In this study, we show that the level of dissolved ketoprofen in the KET-NPs formulation is higher than in the KET-MPs formulation (Figure 1C). Therefore, one pathway is that dissolved ketoprofen on the penetrates the skin, and is transmitted into the blood. On the other hand, our previous reports showed that drug infiltration into skin tissue is enhanced for particles in the size range of approximately 80–200 nm in comparison with drugs in the liquid state, since the skin penetration rate of ketoprofen nanoparticles is higher than for an ointment containing dissolved ketoprofen (commercially available ketoprofen gel ointment, SECTOR gel^®^ 3%) [20]. In addition, we measured the effect of KET-NPs formulation on SC by using fluorescein, and it was not observed the damage in the SC 24 h after application, since the fluorescein infiltration was similar between with or without KET-NPs formulation. This suggests that other pathways may be involved in the skin penetration of ketoprofen nanoparticles, and it has been reported that endocytosis is the major route by which nanomedicines are transported across membranes [26,27,34,35]. Therefore, we investigated the involvement of energy-dependent pathways on the skin uptake of ketoprofen nanoparticles released from the KET-NPs formulation. It has been reported that incubation at a cold temperature (4 °C) inhibits all energy-dependent uptake, including endocytosis, in cells [36]. Our previous reports showed that the amount of penetrated KET increased linearly after the application of either KET-MPs and KET-NPs formulations, but the pharmacokinetic parameters (*J*_c_ and *K*_p_) in the KET-NPs formulation was higher than that in KET-MPs formulation in the normal condition (37 °C) [20]. On the other hand, the pharmacokinetic parameters (*J*_c_, *K*_p_, *K*_m_, *t*_lag_ and *D* values) of the KET-NP formulation under cold temperature conditions (4 °C) were significantly lower than those of the normal condition (37 °C, Table 1). In addition, the amount of KET in the skin tissues of rats receiving the KET-NPs formulation at 4 °C condition was also significantly lower than that of rats receiving the KET-NPs formulation at 37 °C condition (Figure 3C). Furthermore, the decrease in the both drug skin permeation and retention in the KET-NPs formulation under the 4 °C condition were also observed in the SC-removed skin (Figure 3). Those results showed that the penetration of the KET-NPs formulation was significantly inhibited under cold temperature conditions (4 °C) (Figure 3), and energy-dependent uptake is related to the uptake of ketoprofen from the KET-NPs formulation. Moreover, it suggests that the some ketoprofen can penetrate the SC in the nanoparticle state, and the SC-penetrated ketoprofen particles may be uptaken by the energy-dependent pathway. Based on these results, we sought to identify which endocytosis pathways are related to the skin penetration of ketoprofen nanoparticles using inhibitors specific for individual endocytosis pathways (nystatin, dynasore, rottlerin and cytochalasin D) [37,38,39], and found that multi-treatment with nystatin, dynasore and rottlerin prevents the transport of ketoprofen nanoparticles (Figure 4A). In addition, the level of ketoprofen accumulation in the skin was also decreased by multi-treatment with the three inhibitors (Figure 4B). These results suggest that ketoprofen nanoparticles are taken up into cells by three endocytosis pathways (CavME, CME and MP), and cross to the basolateral side, resulting in an enhancement in skin penetration.

**Figure 6:**
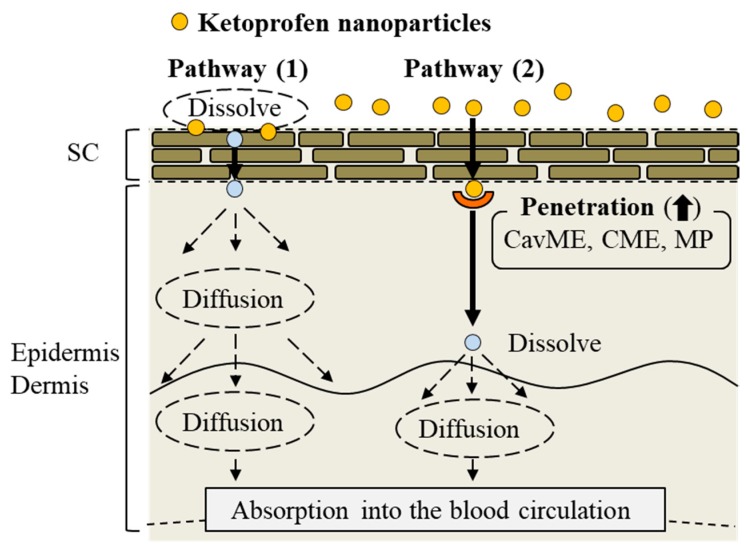
Mechanism for the percutaneous absorption process from the transdermal formulation containing ketoprofen nanoparticles.

It is important to consider the different types of endocytosis activity and the ketoprofen particle size in the KET-NPs formulation. Zhang et al. [40] reported that the size of the vesicles varies with the specific pathway of pinocytosis in the endocytic process, with the sizes corresponding to the CavME, CME, and MP pathways being <80 nm, <120 nm and 100 nm–5 μm, respectively. On the other hand, Yang et al. [41] recently showed that gold nanoparticles taken up by the CavME and CME pathways involved a degradation and transcytosis pathway in the cells. In addition, the ratio of degradation and transcytosis of nanoparticles mediated by MP was lower than for those mediated by CavME and CME in the Caco-2 and HT-29 intestinal epithelial cell lines [41]. In the present study, a ledge comprising individual ketoprofen molecules was observed of the surface of each ketoprofen nanoparticle, and the size frequency of ketoprofen particles in the KET-NPs formulation was approximately 20–200 nm (mean particle size 98 nm) (Figure 1A). Taken together, it is possible that both dissolved ketoprofen on the SC and nanoparticles passed through the SC reflect the skin penetration and accumulation of ketoprofen from the KET-NPs formulation (pathway 1, Figure 6). In addition, the involvement of the CavME, CME and MP pathways are determined by the differences in the size and shape of the nanoparticles, and the contribution to the cellular uptake and intracellular route of the different endocytosis pathways may affect the skin penetration and accumulation of ketoprofen (pathway 2, Figure 6).

These results support previous reports in which endocytosis is the major route by which nanomedicines are transported across membranes [26,27,34,35]. Further studies are needed to clarify the relationships of the three endocytosis pathways and the percutaneous absorption of ketoprofen nanoparticles. Moreover, it is important to elucidate the mechanism of ketoprofen dissolution in the skin penetration process of ketoprofen nanoparticles. Therefore, we are currently investigating the effect of energy-dependent endocytosis on ketoprofen dissolution. Moreover, we are planning to demonstrate the correlation between the activation of the three endocytosis pathways and drug particle size using cultured cells.

## 4. Materials and Methods

### 4.1. Animals

Wistar rats were purchased from Kiwa Laboratory Animals Co., Ltd. (Wakayama, Japan); 7 week-old rats (male, 210–240 g) were used in this study. The rats were housed under normal conditions (7:00 a.m.–7:00 p.m. light, 25 °C), and the water and CE-2 formulation diet (Clea Japan Inc., Tokyo, Japan) were provided freely. The experiments using animals were approved by the animal care and user committee of Kindai University, and carried out in accordance with the Pharmacy Committee Guidelines (project identification code KAPS-25-002, 1 April 2013). In addition, all procedures were in accordance with the Guiding Principles approved by The Japanese Pharmacological Society and with the guidelines for animal experimentation of the International Association for the Study.

### 4.2. Chemicals

Nystatin and carboxypolymethylene (carbopol, Carbopol^®^ 934) were purchased from Sigma-Aldrich Japan (Tokyo, Japan) and Serva (Heidelberg, Germany), respectively. Cytochalasin D, propyl *p*-hydroxybenzoate, isoflurane and commercially available ketoprofen powder (particle size, 7.7 ± 0.28 μm, means ± S.E.) were provided by Wako Pure Chemical Industries, Ltd. (Osaka, Japan). Rottlerin and dynasore were purchased from Nacalai Tesque (Kyoto, Japan) and methylcellulose (MC, SM-4) was obtained from Shin-Etsu Chemical Co., Ltd. (Tokyo, Japan). All other chemicals used were of the highest purity commercially available.

### 4.3. Preparation of the Ketoprofen Transdermal Formulation

Dispersions containing ketoprofen nanoparticles were prepared as follows: ketoprofen powder was added to MC solution in purified water, and subjected to wet milling. The milling (3000 rpm, 30 s × 30 times) was performed by a Bead Smash 12 at 4 °C (Wakenyaku Co. Ltd., Kyoto, Japan) [20,42]. The dispersions containing ketoprofen nanoparticles were mixed with carboxypolymethylene, and the gelled-mixture containing ketoprofen nanoparticles was used as the transdermal formulation containing ketoprofen nanoparticles (KET-NPs formulation). The composition of the KET-NPs formulation was as follows: 1.5% ketoprofen, 0.5 MC, 3% carboxypolymethylene. The particle size and number of ketoprofen particles was measured by a NANOSIGHT LM10 (QuantumDesign Japan, Tokyo, Japan) as follows: viscosity of the suspension, 1.27 mPa∙s; wavelength, 405 nm (blue); time 60 s. Images of the particles were evaluated using a SPM-9700 (Shimadzu Corp., Kyoto, Japan).

### 4.4. Measurement of Ketoprofen by an HPLC Method

The ketoprofen concentration was measured using a Shimadzu LC-20AT system equipped with a column oven CTO-20 A (Shimadzu Corp.). The HPLC conditions were as follows: column, Inertsil^®^ ODS-3 column (3 μm, column size: 2.1 mm × 50 mm, GL Science Co., Inc., Tokyo, Japan); column temperature, 35 °C; detection wavelength, 260 nm; intestinal standard, 1 μg/mL propyl *p*-hydroxybenzoate; the mobile phase consisted of methanol—0.05% trifluoroacetic acid (50:50, *v*/*v*); flow rate, 0.25 mL/min.

### 4.5. Drug Release from KET-NPs Formulations

The release of ketoprofen from the KET-NPs formulation was measured as previously reported using a Franz diffusion cell (reservoir volume 12.2 mL) [20,42]. Briefly, 25 nm- or 450 nm-pore size MF^TM^-MEMBRANE FILTERS (Merck Millipore, Tokyo, Japan) were set in a Franz diffusion cell filled with purified water, and an *O*-ring flange (1.6 cm i.d.) was placed on the filter. Then, 0.3 g of the transdermal formulation was spread uniformly over the filter. The Franz diffusion cell was thermoregulated at 37 °C, and 100 μL of sample solution was withdrawn from the reservoir chamber filled with purified water. The particle size, number and concentration of the ketoprofen in the samples were determined using the NANOSIGHT LM10 and HPLC methods as described above. The area under the ketoprofen concentration-time curve (*AUC*_Release_) was analyzed according to the trapezoidal rule up to the last ketoprofen measurement point (24 h).

### 4.6. In Vitro Transdermal Penetration of KET-NPs Formulation

Skin penetration from the transdermal formulation was measured by using rat abdominal skin set in the Franz diffusion cell filled with purified water [20,42]. The skin was obtained from 7 week-old Wistar rats following the removal of the hair on the abdominal area on the day prior to the experiment. The SC was removed by the tape stripping method as necessary, and the O-ring flange (1.6 cm i.d.) was placed on the skin. Then, 0.3 g KET-NPs formulation (1.5%) was spread uniformly over the skin. In the energy-dependent endocytosis analysis, the Franz diffusion cell was filled with purified water, and thermoregulated at 4 °C, under which condition energy-dependent endocytosis is inhibited [36], or at 37 °C (normal conditions). For the analysis of the different endocytosis pathways by pharmacological inhibitors, inhibitors of CavME (54 μM nystatin) [37], CME (40 μM dynasore) [38], MP (2 μM rottlerin) [39] or phagocytosis (10 μM cytochalasin D) [37] were applied to the removed skin for 1 h prior to the application of the transdermal formulation. The endocytosis inhibitors were dissolved in 0.5% DMSO. The Franz diffusion cell was filled with purified water with or without endocytosis inhibitor, thermoregulated at 37 °C (normal conditions). In this study, 100 μL of sample solution was withdrawn from the reservoir chamber for the measurement of particle size, number and concentration. The size, number and concentration of ketoprofen particles in the samples were determined using the NANOSIGHT LM10 and HPLC methods as described above, and the areas under the penetrated ketoprofen concentration-time curves (*AUC*_Penetration_) were measured by the *AUC* method described above. In addition, the *J*_c_, (penetration rate), *K*_m_ (skin/preparation partition coefficient), *K*_p_ (penetration coefficient through the skin), *D* (diffusion constant within the skin), lag time (*t*_lag_) were analyzed according to Equations (1)–(3) [20]:

(1) $$t_{\mathrm{lag}}=\frac{\delta^{2}}{6D}$$

(2) $$J_{c}=\frac{K_{m}\cdot D\cdot C_{\mathrm{KET}}}{\delta }=K_{p}\cdot C_{\mathrm{KET}}$$

(3) $$Q_{t}=J_{c}\cdot A\cdot \left(t-t_{\mathrm{lag}}\right)$$

The analysis was performed by a nonlinear least-squares computer program (MULTI) [20], and *A* (2 cm^2^), *δ* (0.071 cm) and *Q*_t_ are the effective area of the skin, the thickness of the skin (mean of 6 independent rats), and amount of ketoprofen (*C*_KET_) in the reservoir solution at time *t*, respectively.

### 4.7. In Vivo Percutaneous Absorption of KET-NPs Formulation

The hair on the abdominal area of 7 week-old Wistar rats was removed on the day before the experiment, and 0.3 g KET-NPs formulation (1.5%) was applied to the shaved abdominal skin on the following day. Blood was collected from the right jugular vein via cannulation, and centrifuged (800 g, 20 min, 4 °C) for the measurement of plasma ketoprofen concentrations. The concentration of ketoprofen was determined using the HPLC method described above, and the areas under the plasma ketoprofen concentration-time curves (*AUC*_Plasma_) were evaluated by the *AUC* method described above.

### 4.8. Statistical Analysis

The data are expressed as the mean ± standard error (S.E.) of the mean. Student’s *t*-test was used for two group comparisons, and one-way analysis of variance (ANOVA) followed by Dunnett’s multiple comparison was used for multiple group comparisons. A minimum *p* value of 0.05 (*p* < 0.05) was chosen as the significance level.

## 5. Conclusions

We prepared a transdermal formulation containing ketoprofen nanoparticles 20–200 nm in size since it has been reported that nanoparticles with diameters of 60–100 nm are optimal for the cellular uptake process [17,18,19], and found two pathways, (1) and (2), for skin penetration. Pathway (1) is that dissolved ketoprofen on the SC is penetrates the skin penetration and is transmitted into the blood circulation [20]. For pathway (2), it is hypothesized that ketoprofen nanoparticles are released from the KET-NPs formulation, and reach the underlying epidermis via the SC. After that, the ketoprofen nanoparticles transit into the underlying epidermis and are taken up by three endocytosis pathways (CavME, CME and MP), after which the nanoparticles may dissolve and diffuse into the epidermis and dermis, and resulting, finally, in transition into the blood circulation (Figure 6). These findings provide significant information that can be used to design further studies aimed at developing transdermal delivery systems using nanoparticles.

## Figures and Tables

**Table 1:** Pharmacokinetic analysis of KET-NPs formulation in rat skin at 4 °C and 37 °C.

Parameter	Normal Skin		SC-Removed Skin	
	4 °C	37 °C	4 °C	37 °C
*J*_c_ (nmol·cm^−2^·min^−1^)	26.7 ± 13.5 **	208.7 ± 10.8 *^,^***	28.2 ± 4.6 **	261.4 ± 8.2 *^,^**^,^***
*K*_p_ (×10^−3^·min^−1^)	7.5 ± 2.8 **	59.0 ± 3.1 *^,^***	7.3 ± 0.2 **	74.3 ± 2.5 *^,^**^,^***
*K*_m_ (×10^−3^)	5.5 ± 2.9	4.8 ± 0.8	5.5 ± 0.6	3.4 ± 0.9
*t*_lag_ (min)	8.0 ± 2.2 **^,^***	1.0 ± 0.2 *^,^***	2.2 ± 0.4 *^,^**	0.6 ± 0.1 *^,^***
*D* (×10^−3^·cm^2^·min^−1^)	0.2 ± 0.1 **	1.1 ± 0.3 *^,^***	0.4 ± 0.1 **	1.4 ± 0.5 *^,^***
*AUC*_Penetration_ (μmol·h/cm^2^)	6.1 ± 1.0 **	49.7 ± 6.1 *^,^***	8.8 ± 1.1 **	53.2 ± 5.4 *^,^***

Skin with (normal skin) and without SC (SC-removed skin) was used in this study; the SC was removed by the tape stripping method. The parameter data were analyzed by Equations (1)–(3). *n* = 6. * *p* < 0.05, vs. normal skin at 4 °C for each category. ** *p* < 0.05, vs. normal skin at 37 °C for each category. *** *p* < 0.05, vs. SC-removed skin at 4 °C for each category.

**Table 2:** Effect of endocytosis inhibitors on in vitro transdermal penetration of KET-NPs formulation.

Parameter	Control	Nystatine	Dynasore	Rottlerin	Cytochalasin D	Nys + Dyn + Rot
*J*_c_ (nmol·cm^−2^·min^−1^)	187.9 ± 19.0 **	127.3 ± 0.9 *^,^**	128.5 ± 1.5 *^,^**	128.3 ± 29.5 *^,^**	199.5 ± 26.2 **	27.1 ± 9.3 *
*K*_p_ (×10^−3^·min^−1^)	53.1 ± 5.4 **	35.9 ± 5.5 *^,^**	36.3 ± 9.3 *^,^**	36.3 ± 4.2 *^,^**	56.4 ± 7.3 **	7.2 ± 1.2 *
*K*_m_ (×10^−3^)	3.2 ± 0.9	3.7 ± 0.2	4.2 ± 0.2	2.8 ± 0.8	2.2 ± 0.8	4.3 ± 0.8
*t*_lag_ (min)	0.8 ± 0.3 **	1.3 ± 0.2 *^,^**	1.5 ± 0.2 *^,^**	0.8 ± 0.2 **	0.5 ± 0.2 **	8.0 ± 1.0 *
*D* (×10^−3^·cm^2^·min^−1^)	1.5 ± 0.3 **	0.7 ± 0.1 *^,^**	0.6 ± 0.1 *^,^**	1.4 ± 0.4 **	3.2 ± 1.7 **	0.2 ± 0.1 *
*AUC*_Penetration_ (μmol·h/cm^2^)	50.7 ± 8.7 **	32.9 ± 6.1 *^,^**	35.2 ± 6.3 *^,^**	36.8 ± 7.4 *^,^**	52.6 ± 9.5 **	6.8 ± 2.7 *

The skin samples were co-treated with KET-NPs formulation and inhibitors [0.5% DMSO (control), 54 μM nystatin, 40 μM dynasore, 2 μM rottlerin or 10 μM cytochalasin D]. Nys + Dyn + Rot was showed the multi-treated groups by 54 μM nystatin, 40 μM dynasore, 2 μM rottlerin. The parameter data were analyzed by Equations (1)–(3). *n* = 6–8. * *p* < 0.05, vs. control for each category. ** *p* < 0.05, vs. Nys + Dyn + Rot for each category.

## Abbreviations

ANOVA	one-way analysis of variance
*AUC*	area under the ketoprofen concentration-time curve
carbopol	Carbopol^®^ 934
CavME	caveolae-mediated endocytosis
CME	clathrin-mediated endocytosis
COX	cyclooxygenase enzyme
*D*	penetration coefficient through the skin
*J*_c_	penetration rate
KET-NPs formulation	transdermal formulation containing ketoprofen solid nanoparticles
KET-MPs formulation	transdermal formulation containing ketoprofen microparticles
*K*_m_	skin/preparation partition coefficient
NSAIDs	non-steroidal anti-inflammatory drug
MC	methylcellulose
MP	macropinocytosis
PGs	prostaglandins
SC	stratum corneum
SPM	scanning probe microscope
*t*_lag_	lag time

## References

[1] Malfait A.M., Schnitzer T.J. (2013). Towards a mechanism-based approach to pain management in osteoarthritis. Nat. Rev. Rheumatol..

[2] Voilley N., de Weille J., Mamet J., Lazdunski M. (2001). Nonsteroid anti-inflammatory drugs inhibit both the activity and the inflammation-induced expression of acid-sensing ion channels in nociceptors. J. Neurosci..

[3] Fossgreen J., Ketoprofen (1976). A survey of current publications. Scand. J. Rheumatol. Suppl..

[4] Chi S.C., Jun H.W. (1991). Release rates of ketoprofen from poloxamer gels in a membraneless diffusion cell. J. Pharm. Sci..

[5] Irion G.D., Garrison M.D., Abraham W. (1995). Effect of PGML excipient mixture in a transdermal system on the in vitro transport of estradiol across the skin. Pharm. Res..

[6] Sinh S.K., Durrani M.J., Reddy I.R., Khan M.A. (1996). Effect of permeation enhancers on the release of ketoprofen through transdermal drug delivery systems. Pharmazie.

[7] Cho Y.I., Choi H.K. (1998). Enhancement of percutaneous absorption of ketoprofen: Effect of vehicles and adhesive matrix. Int. J. Pharm..

[8] Sridevi S., Diwan P.V.R. (2002). Optimized transdermal delivery of ketoprofen using pH and hydroxypropyl-β-cyclodextrin as co-enhancers. Eur. J. Pharm. Biopharm..

[9] Podlogar F., Bester Rogac M., Gasperlin M. (2005). The effect of internal structure of selected water–Tween 40^®^–Imwitor 308^®^–IPM microemulsions on ketoprofene release. Int. J. Pharm..

[10] Djordjevic L., Primorac M., Stupar M. (2005). In vitro release of diclofenac diethylamine from caprylocaproyl macrogolglycerides based microemulsions. Int. J. Pharm..

[11] So J.W., Park H.H., Lee S.S., Kim D.C., Shin S.C., Cho C.W. (2009). Effect of microneedle on the pharmacokinetics of ketoprofen from its transdermal formulations. Drug Deliv..

[12] Shinkai N., Korenaga K., Mizu H., Yamauchi H. (2008). Intra-articular penetration of ketoprofen and analgesic effects after topical patch application in rats. J. Control. Release.

[13] Albanese A., Tang P.S., Chan W.C. (2012). The effect of nanoparticle size, shape, and surface chemistry on biological systems. Annu. Rev. Biomed. Eng..

[14] Li Y., Lian Y., Zhang L.T., Aldousari S.M., Hedia H.S., Asiri S.A., Liu W.K. (2016). Cell and nanoparticle transport in tumour microvasculature: The role of size, shape and surface functionality of nanoparticles. Interface Focus.

[15] Shen Z., Nieh M.P., Li Y. (2016). Decorating Nanoparticle Surface for Targeted Drug Delivery: Opportunities and Challenges. Polymers.

[16] Ying L., Wylie S., Tae-Rin L., Sung K., Han M., Dean H., Paolo D., Wing K.L. (2014). Multiscale modeling and uncertainty quantification in nanoparticle-mediated drug/gene delivery. Comput. Mech..

[17] Chithrani B.D., Chan W.C. (2007). Elucidating the mechanism of cellular uptake and removal of protein-coated gold nanoparticles of different sizes and shapes. Nano Lett..

[18] Zhang S., Gao H., Bao G. (2015). Physical Principles of Nanoparticle Cellular Endocytosis. ACS Nano.

[19] Gao H., Shi W., Freund L.B. (2005). Mechanics of receptor-mediated endocytosis. Proc. Natl. Acad. Sci. USA.

[20] Nagai N., Iwamae A., Tanimoto S., Yoshioka C., Ito Y. (2015). Pharmacokinetics and Antiinflammatory Effect of a Novel Gel System Containing Ketoprofen Solid Nanoparticles. Biol. Pharm. Bull..

[21] Gratton S.E., Ropp P.A., Pohlhaus P.D., Luft J.C., Madden V.J., Napier M.E., DeSimone J.M. (2008). The effect of particle design on cellular internalization pathways. Proc. Natl. Acad. Sci. USA.

[22] Youm I., Bazzil J.D., Otto J.W., Caruso A.N., Murowchick J.B., Youan B.B. (2014). Influence of surface chemistry on cytotoxicity and cellular uptake of nanocapsules in breast cancer and phagocytic cells. AAPS J..

[23] Proulx S.T., Luciani P., Dieterich L.C., Karaman S., Leroux J.C., Detmar M. (2013). Expansion of the lymphatic vasculature in cancer and inflammation: New opportunities for in vivo imaging and drug delivery. J. Control. Release.

[24] Qin L., Zhang F., Lu X., Wei X., Wang J., Fang X., Si D., Wang Y., Zhang C., Yang R. (2013). Polymeric micelles for enhanced lymphatic drug delivery to treat metastatic tumors. J. Control. Release.

[25] Zhang X., Hu W., Li J., Tao L., Wei Y. (2012). A comparative study of cellular uptake and cytotoxicity of multi-walled carbon nanotubes, graphene oxide, and nanodiamond. Toxicol. Res..

[26] Wang J., Byrne J.D., Napier M.E., DeSimone J.M. (2011). More effective nanomedicines through particle design. Small.

[27] Rappoport J. (2008). Focusing on clathrin-mediated endocytosis. Biochem. J..

[28] Benmerah A., Lamaze C. (2007). Clathrin-coated Pits: Vive la différence?. Traffic.

[29] Medina-Kauwe L.K. (2007). “Alternative” endocytic mechanisms exploited by pathogens: New avenues for therapeutic delivery?. Adv. Drug Del. Rev..

[30] Swanson J.A. (2008). Shaping Cups into Phagosomes and Macropinosomes. Nat. Rev. Mol. Cell Biol..

[31] Swanson J.A., Watts C. (1995). Macropinocytosis. Trends Cell Biol..

[32] Tamaru M., Akita H., Fujiwara T., Kajimoto K., Harashima H. (2010). Leptinderived peptide; a targeting ligand for mouse brain-derived endothelial cells via macropinocytosis. Biochem. Biophys. Res. Commun..

[33] Bhattacharya S., Roxbury D., Gong X., Mukhopadhyay D., Jagota A. (2012). DNA conjugated SWCNTs enter endothelial cells via Rac1 mediated macropinocytosis. Nano Lett..

[34] Longfa K., Jin S., Yinglei Z., Zhonggui H. (2013). The endocytosis and intracellular fate of nanomedicines: Implication for rational design. Asian J. Pharm. Sci..

[35] Aderem A., Underhill D.M. (1999). Mechanisms of phagocytosis in macrophages. Annu. Rev. Immunol..

[36] He Z., Liu K., Manaloto E., Casey A., Cribaro G.P., Byrne H.J., Tian F., Barcia C., Conway G.E., Cullen P.J. (2018). Cold Atmospheric Plasma Induces ATP-Dependent Endocytosis of Nanoparticles and Synergistic U373MG Cancer Cell Death. Sci. Rep..

[37] Mäger I., Langel K., Lehto T., Eiríksdóttir E., Langel U. (2012). The role of endocytosis on the uptake kinetics of luciferin-conjugated cell-penetrating peptides. Biochim. Biophys. Acta.

[38] Malomouzh A.I., Mukhitov A.R., Proskurina S.E., Vyskocil F., Nikolsky E.E. (2014). The effect of dynasore, a blocker of dynamin-dependent endocytosis, on spontaneous quantal and non-quantal release of acetylcholine in murine neuromuscular junctions. Dokl. Biol. Sci..

[39] Hufnagel H., Hakim P., Lima A., Hollfelder F. (2009). Fluid phase endocytosis contributes to transfection of DNA by PEI-25. Mol. Ther..

[40] Zhang S., Li J., Lykotrafitis G., Bao G., Suresh S. (2008). Size-dependent endocytosis of nanoparticles. Adv. Mater..

[41] Yang D., Liu D., Qin M., Chen B., Song S., Dai W., Zhang H., Wang X., Wang Y., He B. (2018). Intestinal Mucin Induces More Endocytosis but Less Transcytosis of Nanoparticles across Enterocytes by Triggering Nanoclustering and Strengthening the Retrograde Pathway. ACS Appl. Mater. Interfaces.

[42] Nagai N., Tanino T., Ito Y. (2016). Pharmacokinetic Studies of Gel System Containing Ibuprofen Solid Nanoparticles. J. Oleo Sci..

